# Supplementary material for: An artificial intelligence life cycle: From conception to production
Source: Patterns (N Y). 2022 Apr 13;3(6):100489. doi: 10.1016/j.patter.2022.100489 (PMC9214328; doi:10.1016/j.patter.2022.100489)
Supplement: Document S1. Tables S1 and S2 [file mmc1.pdf]

Patterns, Volume *3*

Supplemental information

An artificial intelligence life cycle:

From conception to production

Daswin De Silva and Damminda Alahakoon

Table S1: Application of the CDAC AI Lifecycle for Conception to Production of a Micro-Profiling Service for a Large Energy Retailer

**Stages of the AI Lifecycle**	**Conception to production of a micro-profiling service for a large energy retailer**
Identify and formulate the problem	Energy consumption based on lifestyle and generation using rooftop solar and battery storage has created a diversity of energy consumers that cannot be classified into simple tariffs and plans.<br>How can an AI be used to identify micro-profiles that encapsulate energy usage patterns, generation patterns and lifestyle factors influencing both aspects so that a more comprehensive and inclusive tariff structure can be developed?
Review data and AI ethics	Preserve privacy and confidentiality of energy consumers and the data they have shared with the retailer. Ensure identifying customer information is excluded from the final micro profiles generated. Seek ethics approval from the organisational ethics committee or submit a report to the regulatory body for the sector.
Review technical literature on AI algorithms, applications and pre-trained models	Review and evaluate all segmentation and clustering algorithms, specifically focusing on those applied in customer profiling applications. Consider the use of pre-trained models for demographics and geographic profiling
Data preparation	Conduct a data/ database audit of all customer information recorded within the organisation, such as demographic, behavioural, psychometric, transactions, consultations, feedback data.
Data exploration	Evaluate the data in terms of attributes, records, data quality measures and relevance to the expected solution. Use visualisations, statistical techniques, correlation analysis and sampling methods.
External data acquisition	In consultation with domain experts assess the quality and quantity of data that they would be using to profile customers for other intentions, such as sales, marketing or customer relationship management. Explore options for collecting more nuanced demographic and lifestyle information about the target segments serviced by the retailer.
Data pre-processing	Apply relevant pre-processing techniques such as missing value imputation, range/format checks, transformation of categorical and textual data into numerical format that contain the appropriate semantics for the task at hand
Build initial AI model	Start with the least complex segmentation / clustering algorithm that can accommodate the volume of data that has been collected for this exercise. Determine a number of levels of profiles to be generated, define what is meant by 'micro' for this segmentation algorithm.
Data augmentation	Consider the use of externally sourced data or integrated/ fused data sequences such as consumption and generation patterns combined, or lifestyle factors and suburb profiles fused together.
Develop a benchmark	The current profiling scheme can be directly used as a benchmark to evaluate the micro profiles, or the profiles recommended by a human expert can also be used.
Build multiple AI models	Build new models in increasing algorithmic complexity, also consider including all data dimensions or combinations of dimensions

Evaluate primary metrics	Starting with the standard metrics, accuracy, precision, recall and F1 score, consider developing metrics that incorporate domain intricacies
AI model explainability	Utilise XAI methods and libraries to draw meaning and causality from the generated micro profiles, starting with the attribute densities and developing into combinatorial factors.
Evaluate secondary metrics	Computational performance and use of memory in re-segmentation and also mapping new data into one or more micro profiles.
AI model deployment and risk assessment	Deploy the model on a suitable infrastructure specification that supports multiple end-users and use cases.<br>Determine the risks of misclassification, noise, excess segmentation and low value segments that do not contribute to tariff design
Post-deployment review	Compliance, standardisation, post-implementation documentation, as well as service level agreements for model use and maintenance
Operationalise using AI pipelines	Utilise a microservices platform to scale up the deployment of profiles to service different use cases
Hyperautomation of processes and systems	Link up the profiles to automated services such as billing, invoicing, customer experience, planning and budgeting
Monitor and evaluate performance	Monitor performance metrics for all use cases when deployed and continuous evaluation to suit evolving needs and expectations.

Table S2: Application of the CDAC AI Lifecycle for Conception to Production of an AI Conversational Agent for Patient-Centred Healthcare

**Stages of the AI Lifecycle**	**Conception to production of an AI conversational agent (chatbot) for patient-centred healthcare**
Identify and formulate the problem	Increasing smartphone ownership and widespread use of the Internet to find health-related information has created an organisational void where individuals prefer unverified online resources over formal healthcare services for informational support and emotional support.<br><br>How can an AI chatbot be used to address this organisational void by providing personalised, intuitive, patient-centred support and information that is structured and approved by healthcare practitioners?
Review data and AI ethics	Healthcare regulation and medical ethics ensure privacy and confidentiality of patient and clinical data, with access being limited to approved individuals, applications and activities.<br><br>When investigating the potential of AI for addressing this organisational void, AI, data, patient ethics approval must be sought from an appropriate body and a representative terms and conditions must be presented to the end-user before they engage with the AI chatbot. The preference should be towards complete anonymity where no identifying information is collected by the AI service.
Review technical literature on AI algorithms, applications and pre-trained models	Review and evaluate proprietary and open-source platforms for building out a chatbot, such as Google Dialogflow, Azure bot service, Rasa. Review and evaluate pre-trained language models that can be plugged into a conversational service, as well as suitable prediction, classification and segmentation algorithms.
Data preparation	Conduct a data/ database audit of all conversational records available within the organisational systems. Determine the data structures and models used and attempt to integrate these into a unified structure that is fit for purpose.
Data exploration	Evaluate the data in terms of attributes, records, data quality measures and relevance to the expected solution. Use visualisations, statistical techniques and sampling methods.
External data acquisition	In consultation with domain experts (healthcare practitioners), assess the quality and quantity of conversational records for building out an intelligent chatbot service. If insufficient explore third party datasets provided by the chatbot platform providers and other research groups or organisations working in this space.
Data pre-processing	Apply relevant natural language pre-processing techniques (such as abbreviated terms, medical terms, emojis, short form sentences) that

	will structure and normalise the datasets to suit the conversation training process.
Build initial AI model	Explore several configurations for the AI capabilities of a chatbot, such as classification of end-user inputs into tasks or intents, prediction of the next question or comment by the end-user, using segmentation output to determine what type of end-user and what kind of health services might be required (given that the end-user is anonymous and no exchange of identifying data), optimise the recommended self-help or healthcare advice provided in terms of the emotional or informational needs expressed.
Data augmentation	For the four AI capabilities, assess the effectiveness of all the initial models and then explore opportunities for augmentation such as, more training data for selected parts of a conversation, dynamic time warping to align intents and actions required from the chatbot
Develop a benchmark	Quantify the level of accuracy expected from a human operator, along the scale of a beginner to an expert. i.e. know how many questions or comments will be misunderstood in the first round, second attempt and third.
Build multiple AI models	Explore more configurations of the chatbot information flow, also varying the algorithms and parameters used for each sub task
Evaluate primary metrics	Starting with the standard metrics, accuracy, precision, recall and F1 score, consider developing metrics that incorporate domain intricacies
AI model explainability	Develop the capacity to explain and interpret as a sequence of words or phrases why and how a certain response was generated.
Evaluate secondary metrics	Computational performance and use of memory for conversations of varying length and depth, taking into account repeat questions and follow-up clarification questions
AI model deployment and risk assessment	Deploy the model on a suitable infrastructure specification that supports multiple end-users without any interruptions or performance issues.<br><br>Determine the risks of drop-outs, incorrect responses, turnaround time for direct human involvement as well as the boundaries of conversation.
Post-deployment review	Compliance, standardisation, post-implementation documentation, and small-scale clinical trials of the effectiveness of the chatbot in diverse healthcare settings
Operationalise using AI pipelines	Utilise a microservices platform to scale up the deployment of the chatbot to serve many individuals across multiple healthcare functions

Hyperautomation of processes and systems	Link up the chatbot to consultation, referral and post-treatment/surgery processes
Monitor and evaluate performance	Monitor performance metrics for every engagement when deployed and continuous evaluation to suit evolving needs and expectations in the healthcare domain.
