# Supplementary material for: An artificial intelligence life cycle: From conception to production
Source: Patterns (N Y). 2022 Apr 13;3(6):100489. doi: 10.1016/j.patter.2022.100489 (PMC9214328; doi:10.1016/j.patter.2022.100489)
Supplement: Document S2. Article plus supplemental information [file mmc2.pdf]

Descriptor

Patterns

An artificial intelligence life cycle: From conception to production

Highlights

- The CDAC AI life cycle is a comprehensive approach from conception to production of AI
- It begins with risk assessment, followed by design, develop, and deploy phases
- The technical details of 19 constituent stages of the life cycle are articulated
- A taxonomy of AI capabilities and the organizational context of AI are presented

Authors

Daswin De Silva, Damminda Alahakoon

Correspondence

d.desilva@latrobe.edu.au

In brief

Artificial intelligence (AI) is embedded in our way of life, transforming how we live and work. AI is enabling new opportunities for value creation in organizations, industries, and society. Collectively for commercial, social, and national interests, as well as all corresponding stakeholders, it is vital to know, apply, adapt, and even critique an inclusive, evidence-based approach to conceptualize, assess risks, design, develop, and deploy an AI solution. This article presents the CDAC AI life cycle, from conception to production of AI.

De Silva & Alahakoon, 2022, Patterns *3*, 100489
June 10, 2022 © 2022 The Authors.
https://doi.org/10.1016/j.patter.2022.100489

Patterns

Descriptor

An artificial intelligence life cycle: From conception to production

Daswin De Silva[1,2,*] and Damminda Alahakoon[1]
[1]Centre for Data Analytics and Cognition (CDAC), La Trobe University, Bundoora, VIC, Australia
[2]Lead contact
*Correspondence: d.desilva@latrobe.edu.au
https://doi.org/10.1016/j.patter.2022.100489

THE BIGGER PICTURE Artificial intelligence is enabling new opportunities for value creation in organizations, industries, communities, and overall society. However, exponential commercial investments in the private sector should not overlook the public interest and social value of this emerging technology powerhouse. The world's superpowers are also competing for leadership in AI and the contentious pursuit of artificial general intelligence. Collectively for commercial, social, and national interests, as well as internal and external stakeholders, it is vital to know, observe, understand, apply, adapt, and even critique an inclusive, evidence-based approach to conceptualize, assess risks, design, develop, and deploy an AI solution. This article presents the CDAC AI life cycle, a comprehensive approach that addresses and accounts for all challenges from conception to production of AI.

1 2 3 4 5 Mainstream: Data science output is well understood and (nearly) universally adopted

SUMMARY

This paper presents the "CDAC AI life cycle," a comprehensive life cycle for the design, development, and deployment of artificial intelligence (AI) systems and solutions. It addresses the void of a practical and inclusive approach that spans beyond the technical constructs to also focus on the challenges of risk analysis of AI adoption, transferability of prebuilt models, increasing importance of ethics and governance, and the composition, skills, and knowledge of an AI team required for successful completion. The life cycle is presented as the progression of an AI solution through its distinct phases—design, develop, and deploy—and 19 constituent stages from conception to production as applicable to any AI initiative. This life cycle addresses several critical gaps in the literature where related work on approaches and methodologies are adapted and not designed specifically for AI. A technical and organizational taxonomy that synthesizes the functional value of AI is a further contribution of this article.

INTRODUCTION

Despite the advances, existing work on methodologies and life cycles for artificial intelligence (AI) initiatives do not provide complete coverage from conception to production and are limited in the level of technical detail, workforce requirement, and administrative responsibilities of individual phases. Partridge was one of the first researchers to propose an AI methodology that intertwines the capabilities of software engineering solutions and the expectations of AI models.[1] Drawing on the distinction between AI problems and conventional software engineering, he posited that AI constructs an "adequate approximation," in contrast to a complete solution in standard software engineering, through the discovery and elimination of observed behavioral inadequacies. Depicting the resurgence of AI and machine learning as the more practice-oriented disciplines of data mining and computational intelligence, the Cross-Industry Standard Process for Data Mining (CRISP-DM)[2] gained wide recognition in academia and industry as a *de facto* standard for the use of AI in decision-making and practical problem solving. CRISP-DM consists of six phases: business understanding, data understanding, data preparation, modeling, evaluation, and deployment. Drawing on the success of CRISP-DM, several variations, such as automation focused,[3] geo-located teams,[4] contextual diversity,[5] and knowledge repositories,[6] as well as adaptations of CRISP-DM for emerging disciplines, such as big data,[7]

This is an open access article under the CC BY license (http://creativecommons.org/licenses/by/4.0/).

cybersecurity,[8] and fintech,[9] have been proposed. More recently, when the terminology transitioned from data mining to "data science" and "data analytics," the Team Data Science Process (TDSP)[10] and the Microsoft best practices approach[11] have been popularized in place of CRISP-DM. TDSP is presented as a life cycle that can be used to structure a data science project that utilizes machine learning algorithms or predictive analytics techniques for solution development. TDSP is also interpreted as a cross between the SCRUM framework and CRISP-DM. The Microsoft best practices approach summarizes three key findings as best practices for AI projects. They are the importance of an accurate data discovery and management process, constraints on customization and re-use for different use cases, and the lack of modularity of AI components, which necessitates the entire team to work closely on all modules.

RESULTS

Drawing on this context of life cycles and methodologies, we have identified that CRISP-DM,[2] TDSP,[10] and the Microsoft best practices model[11] are the industry and academic baselines that are being "adapted" for contemporary AI projects. This in itself demonstrates the need for a life cycle approach that has been conceived exclusively to address the challenges of designing, developing, deploying, and managing an AI solution. The initial distinction followed by the consolidation of data, algorithms, and systems when building an AI solution, as well as a sufficiently technical level of detail of each step of an approach, have not been deliberated in related work. Although AI solutions are a team effort, the responsibilities and the expertise required of these team members have not been articulated. The currency of pre-trained models, third-party code repositories, AI ethics, and governance frameworks have not been outlined as part of a development life cycle or methodology. Extending from ethics and governance, an overarching systems view of AI adoption that identifies and assesses preliminary risks across the design, development, and deployment stages, as well as each individual phase, is a further shortcoming. Despite these deficiencies and the absence of a unifying life cycle, the impact, influence, and thereby importance of AI across national, social, economical, and personal interests continues to grow exponentially.

In this paper, we address these drawbacks by presenting and articulating (1) the preliminary risk assessment for identifying and evaluating organizational and system level risks in the adoption of AI and (2) the CDAC AI life cycle that characterizes the design, development, and deployment of AI systems and solutions. The CDAC AI life cycle further enables continuous, multi-granular expansion of the overarching preliminary risk assessment through its constituent stages and phases. CDAC is the acronym of our research center, Center for Data Analytics and Cognition, as this life cycle is informed by our experience and expertise at CDAC of more than a decade of AI, across academic research, technology development, industry engagement, postgraduate teaching, doctoral supervision, and organizational consultancy. Some highlights from our recent work are Bunji, an empathic chatbot for mental health support;[12] solar nowcasting for optimal renewable energy generation;[13] robust multi-step predictor for energy markets;[14] unsupervised learning with vector symbolic architectures;[15] emotions of COVID-19 from self-reported information;[16] machine learning for online cancer support;[17,18] self-building AI for smart cities;[19] intelligent driver behavior change detection;[20] an incremental learning platform for smart traffic management;[21] and a reference architecture for industrial applications of AI.[22] We anticipate that our contribution will create awareness, instill knowledge, and stimulate discussion and debate that will inform research, applications, and policy developments of AI for humanity.

Preliminary risk assessment

The increasing sophistication and embedding of AI in all forms of digitalized systems and services, including organizational, social, economical, and governmental, necessitates an overarching risk assessment before its adoption. Even in settings where the selected AI capability has matured and established in other sectors, it still needs to be socialized and assessed for the local setting and its environment, focusing on people, processes, platforms, and relevant external factors. This risk assessment is distinguished from the risk review and analysis conducted within the life cycle, specifically "stage 2—review data and AI ethics" and "stage 15—AI model deployment and risk assessment," because here, the scope is at the systems level, focusing on the risks associated with the key aspects of privacy, cybersecurity (information and technology security), trust, explainability, robustness, usability, and social implications of these aspects. The evaluation and defense against vulnerabilities of AI applications have been deliberated in recent work,[23–25] in the same context of addressing high-level risk factors from conception to production and across the entire life cycle. A general synopsis of each aspect is presented below. However, this list is not exhaustive, as there can be other risk factors at the systems level that are specific to the domain of application, for instance, risk factors associated with formal methods software engineering of mission critical systems in the energy sector or national security, and regulatory requirements of software-based medical devices in the healthcare domain.

Privacy. The risk assessment of privacy should consider (1) the impact of AI adoption on the privacy of all systems and data accumulated in these systems; (2) the levels of privacy to be administered on each system, data point, and data linkage during AI adoption and development; (3) the impact of AI capabilities and diverse types of machine learning, such as unsupervised, transfer, and federated learning on privacy preservation; (4) privacy of the storage and staging of data during AI model design and development; and (5) privacy when AI models are being used in organizational processes and decision making.

Cybersecurity. The risk of cybersecurity should be assessed in equal importance to the impact it can have on the foundational and source systems that AI leverages for input and output. Cyber threats and attacks can impact all 19 stages of the AI life cycle; thus, the same rigorous risk assessment that applies to organizational assets of information and technology must be extended to the AI project and its outcomes, across the three phases of design, development, and deployment. A summary comparison of information security risk analysis methods[26] and more recent resource-based approaches for cybersecurity risk management[27] (in contrast to the conventional compliance-based methods) are enabling this expansion from ICT systems view

CellPress
OPEN ACCESS

toward the stages of the AI life cycle and the outcomes of each stage.

Trust, interpretability, explainability, and robustness. These aspects are rigorously assessed within AI ethics frameworks. Ethics frameworks are discussed in "stage 2—review data and AI ethics," specifically the Ethics Guidelines published by the European Union's High-Level Expert Group, which is entitled "Trustworthy AI," but also deliberates traceability, interpretability, explainability, and robustness factors. In contrast to "stage 2—review data and AI ethics," the scope of this risk assessment should be at systems level and not at the level of AI models. For instance, with interpretability the main considerations include integrating interpretability into the design phase for applications that require intrinsically interpretable models (contrast this with explainable AI techniques that can only be applied after the AI model is fully developed), the levels of interpretable detail required, and the computational performance of interpretation from real-time to batch mode. Similarly, robustness entails the need for consistent and rigorous practice across data collection (independent and identically distributed variables), augmentation of anomalies and data perturbations, the learning process, use of pre-trained models, evaluation metrics, and the uptake of AI solutions. Robustness in model development, evaluation, and deployment phases is more widely practiced, and we discuss these in the corresponding stages of the life cycle. Trust also spans across data collection (trustworthy source systems and external data feeds), data augmentation (trustworthy techniques for addressing data imbalance issues), and the training, skills, and organizational change factors to ensure that the uptake of AI is sustainable.

Usability. The risk assessment of usability should focus on the skills and capabilities of the employees in the organization, as well as how receptive they will be toward the introduction of AI models into organizational systems and processes. If the uptake is low, then the risk of loss is greater and should be evaluated in terms of other benefits of AI adoption. Upgrades and updates to existing systems for handling the scale and scope of data and computation required by AI models is another consideration that needs to be assessed before the initiation of an AI project. The integrity of AI is assured through the re-implementation of algorithms and models; however, this must be offset against risks to usability due to revised model parameters and unfamiliar interfaces.

Social implications. All risk factors must be assessed in terms of the end-users (or consumers) of the AI models, as well as the actions and decisions prescribed by the AI. Both positive and negative impact of the AI solution on all sociodemographic segments of society, from the affluent to the marginalized and disadvantaged, should be evaluated and documented as part of this preliminary risk assessment.

Although termed "preliminary," the same risks can be assessed during the application of the life cycle where each risk factor is reviewed and reassessed within the context of the stages of the life cycle. This review can be conducted in the form of a risk matrix[28] that articulates each risk factor in terms of the harm severity and probability for each of the 19 stages. The completeness of identifying, analyzing, and reviewing risk factors at the preliminary (or abstract) level of AI adoption and the intricate level of each stage of the AI life cycle, ensures that the AI initiative is fit for purpose, sustainable, and also forms a blueprint for future AI endeavors.

The CDAC AI life cycle

Figure 1 illustrates the complete AI life cycle, where the shaded parallelograms represent the three phases: (1) design, (2) develop, and (3) deploy. Each phase requires specific human expertise, which are also depicted in the same figure, as design (AI/data scientist), develop (AI/ML scientist), and deploy (AI/ML engineer). The AI/data scientist tasked with the design phase is typically a senior role with several years of experience. They should be able to formulate the problem and then conceptualize a solution drawing on existing literature and their past experiences of working across diverse AI projects. They should also be able to identify the representative data, required data, and available data, by working through the first five stages of the life cycle, when they hand over a prescriptive problem formulation, solution description, and representative data to the AI/ML scientist responsible for the develop phase. This AI/ML scientist is typically a junior role that is more technical and less conceptual, with in-depth technical expertise in AI algorithms, model development, and evaluation. They will work through the next seven stages to transform the problem formulation into a prototypical AI model. Finally, in the deploy phase, an AI/ML engineer further transforms this prototypical AI model into a deployed service or solution that is standardized for access by all stakeholders and end-users. The AI/ML engineer is typically from the DevOps domain. DevOps being a mature practice in software development and IT operations, the skills required for this phase are common, but they need to be consolidated with knowledge and experience in the nuances of deploying AI models. The AI/ML engineer will work through the final seven stages to deliver an AI solution that is part of a larger process and can be automatically monitored across several metrics for quality and accuracy. Depending on the size, scale, and scope of the project, multiples of these roles may need to be contracted or recruited. In conjunction with these primary technical roles, an ethicist (or ethics committee), a project manager, a pool of domain experts, a participatory design group, a pilot study cohort, legal counsel on IP law, and a steering/advisory committee with full oversight are secondary enabling roles that add value, inclusivity, and quality to the AI endeavor. In the following subsections, the 19 stages of the life cycle are described. The execution of all 19 stages depends on the type of project, project timelines, organizational data maturity, and AI expertise. Even where a subset of the stages are undertaken, it is imperative that all stages are given due consideration and formally documented for successful completion of an AI project.

Identify and formulate the problem

The life cycle begins with the identification, elucidation, and formulation of the problem, in terms of its typology, the environment (or setting), the expected objective, people (or stakeholders), systems, processes, and data. Typology can be broadly classified into strategic, tactical, operational, and research. The strategic typology takes a high-level transformative approach of leveraging AI to address a number of challenges in a specific topic, domain, or area of interest, such as "How can AI be used to address the challenges impacting healthcare services during a global pandemic?" or "How can AI be leveraged in

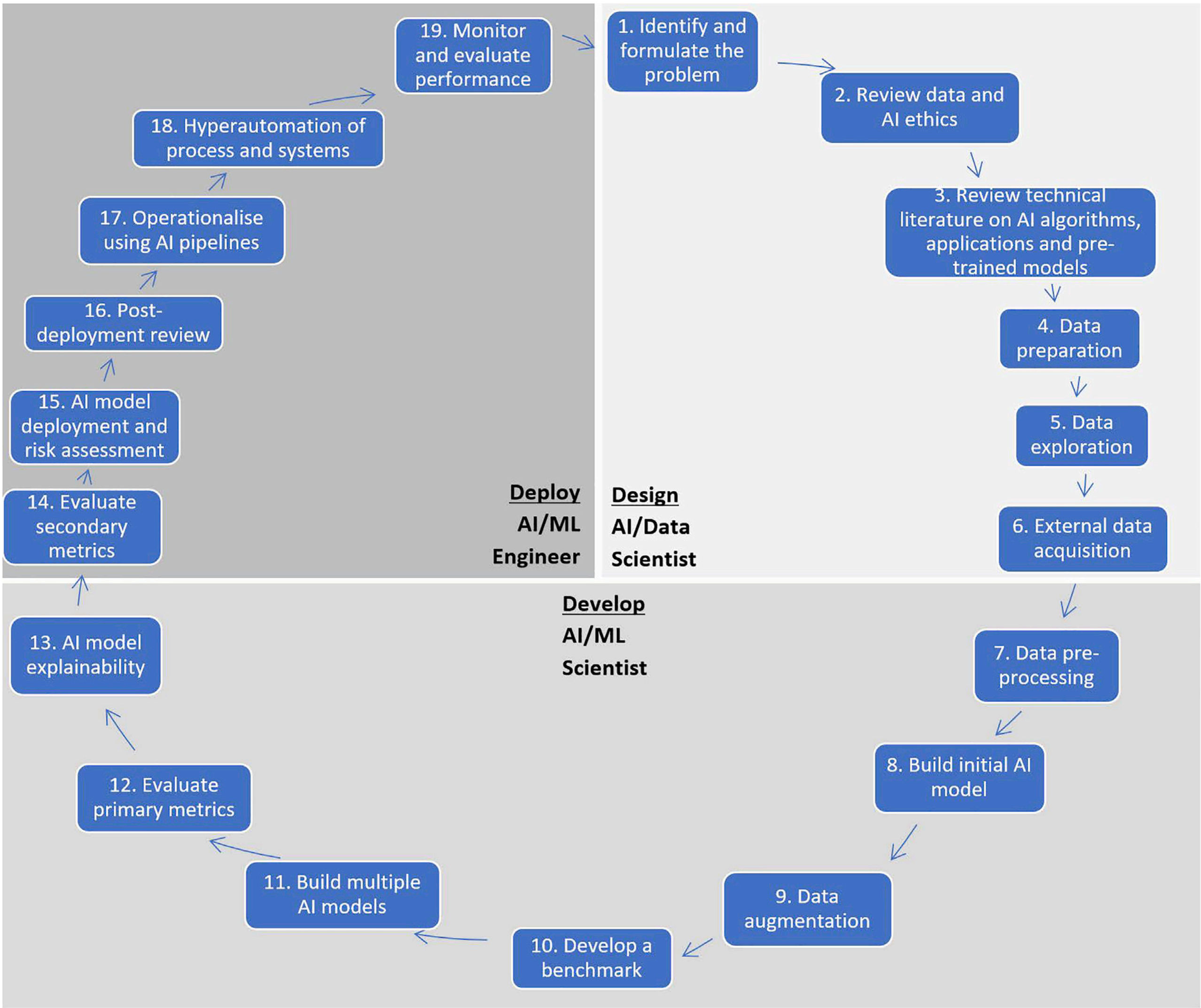

Figure 1. The CDAC AI life cycle: Three phases of (1) design, (2) develop, and (3) deploy and 19 stages

the energy sector to achieve net zero carbon emissions?" The tactical typology is more goal oriented and presents itself as an advancement over the current manual, mechanical, technical, or computational approach to solving the problem or addressing challenges associated with the problem, such that AI augments productivity or quality of outputs. For instance, "using AI to increase market share or become market competitive" or "reducing the turnaround time of an assembly line by a factor of *n*." Thirdly, an operational typology signifies the direct application, implementation, or replication of an AI capability that has already been validated in a same/similar problem, setting, or domain. Examples of an operational typology are, "using AI to forecast weekly hospitalizations during a disease outbreak" and "using AI to profile energy consumers and develop a micro-segmentation strategy for usage optimization." Finally, the research typology focuses on algorithmic novelty that surpasses the current state-of-the-art in AI for solving a collection of similar or related problems, such as "using transformers for increased accuracy of predictions" or "using swarm intelligence to build digital twins." To accommodate and explore this diversity of typology, organizations and team leaders must consult or collaborate with external parties with AI expertise. These discussions must be driven by a shared focus and common vocabulary that enables unbiased evaluation of feasibility, value, and impact. Alongside the next phase, these discussions should also include, alternate solutions that are model-based or non-intelligent but highly computational, budgets, planning, risk assessments, and downstream implications, such as ethics and societal considerations. The setting of the problem formulation influences the objective, such as commercial settings where the focus is monetary, increased revenue, reduced cost, or increased customer value. In an industrial setting, the focus would be on productivity, process efficiencies, or process transformation, and, in a research setting, the objective should be closely aligned to solving or enabling solutions to the research question through one or more primary AI capabilities: prediction,

classification, association, or optimization. The current manual or systems-driven solution to the problem provides the context for its formulation, considering sequential and parallel tasks, primary and alternate flows of information, and rules and policies within the organization. Finally, the "digital representations" of the problem formulation must be identified in terms of data models, data repositories, data owners/stewards, source systems, data governance, ethics frameworks, and metadata.

Review data and AI ethics

In this stage, the problem formulation, selected approach, potential solution, and required datasets must be cross-examined and validated for potential security risks (linking back to the preliminary risk assessment), and ethical and legal conformity. Given that the AI team possesses a highly technical skill set, this review of ethics must be conducted by professional ethicists with the required background in both theory and application. It is recommended to start with a broad-based ethics framework that narrows down to the granular level of the problem setting, algorithmic justice (across gender, ethnicity, sexuality, and other minorities),[29,30] data representations, and stakeholder interests. In scrutinizing the societal implications of an AI project, it is further recommended that a participatory design approach is adopted to engage representative individuals and communities that are likely to be impacted by the AI model and its decision outcomes.[31,32] As of 2019, 84 AI ethics frameworks have been proposed,[33] with increasing usage and prominence toward the IEEE Global Initiative on Ethics Aligned Design[34] and the Ethics Guidelines published by the European Union's High-Level Expert Group on AI.[35] The latter has been further expanded into a regulation for selected applications of AI.[36] This diversity of frameworks and abundance of vague principles and best practices is a further complexity that needs to be addressed by the ethicists and the AI experts together in finding the optimal balance of AI effectiveness and ethical practice. Projects classified in the operational typology where the AI capability has already been implemented elsewhere may not require an ethicist input as this information is already formally documented and widely accepted, such as credit scoring and its derivatives, which is one of the first applications of AI that has now reached peak maturity.

Review technical literature on AI algorithms, applications, and pre-trained models

The problem formulation provides necessary context to explore and review published research, deployed systems, solutions, and libraries that have been applied in similar settings. Some of the more frequently used information sources, such as search engines for research articles (e.g., Google Scholar), publishing platforms (e.g., Medium), Q&A sites (e.g., Stack Exchange), code repositories (e.g., GitHub), cloud platform providers (e.g., Azure, AWS, GCP), and social media (e.g., Twitter). The types of resources are expansive and include literature reviews, commentaries, letters, articles, op-ed, case studies, best practices, product/tool documentation, tutorials, demonstrations, and API documentation, as well as responses, upvotes, and likes on Q&A forums. Furthermore, recent developments in open access pre-trained AI models, such as AlexNet,[37] ResNet,[38] BERT,[39] and GPT,[40] should be studied to understand and explore how they can be re-purposed, retrained, or fine-tuned, instead of building a new AI model from scratch.

When deciding to use pre-trained models, several other factors need to be considered. They are, licensing, model fit for purpose, disparity between pre-trained setting and application setting, and recent developments or data excluded by the model. On matters of licensing, most pre-trained models are open source or public domain to encourage further research and application that validate or advance the model; however, the underlying training data may carry a different license altogether or be limited in use for research endeavors, prohibiting commercial applications.[41,42] In such instances, legal counsel must be sought to ensure that consent, permission, or constraints are implemented and adhered to during the course of the project.

Data preparation

Most often, the data sources identified during problem formulation have been accumulated organically, do not conform to a unified structure, and exist in silos. For example, patient data (or even customer data) are generally distributed across numerous dimensions, such as demographic, behavioral, psychometric, transactions, consultations, or feedback, and these dimensions are accumulated in distinct source systems, at varied times, by different individuals.

It is typically not recommended to use siloed data for building AI models, as this creates an inherent data bias that will propagate through to the AI model, its outcomes, and its generalizability to new data. The recommended approach is to design and develop a unified data repository, such as a data warehouse,[43,44] data lake,[45] or data lakehouse,[46,47] that centralizes data access, ownership, stewardship, metadata, data ethics, governance, and regulations, before the development of AI models.

However, there can be well-defined problems where siloed data with a narrow focus are necessary to ensure model effectiveness or to satisfy data privacy and confidentiality constraints. Also, not every AI project has the timeline or budget for a unified data structure, which makes this an acceptable risk that must be clearly specified and documented. A more recent solution to the siloed data challenges is to use AI itself to develop a meta-layer or representation of all the data sources, structured and unstructured, using techniques such as federated learning,[48,49] representation learning,[50,51] and latent learning.[52,53]

Data privacy, confidentiality, and integrity are related to data preparation, but not directly applicable to the AI life cycle. Also discussed as the cybersecurity triad, in terms of data, these topics are typically regulated by law. The General Data Protection Regulation is the gold standard across the world for data protection and regulation.[54] Furthermore, datasets and sources must be assessed for security risks, such as poisoning attacks and backdoor attacks.

Data exploration

This stage focuses on the actual data, in contrast to the previous stage where the focus is the structure of data. The stage typically begins with a comparison with industry benchmarks and algorithmic baselines reported in the literature, where similar problems have been addressed. The data structure generated in the preparation phase is populated with actual data, using techniques such as data visualization, correlation analysis, aligning data granularity, checking relationships between data points and attributes, handling outliers, and enforcing data quality

checks. Data visualization entails pairwise plots, projections of the entire dataset, and building interactive dashboards, all of which enable the AI team to scrutinize the dataset for accuracy, relevance, quality, and volume. Multicollinearity and high-correlated attributes are usually removed at this stage, but occasionally they are labeled and preserved for further exploration in subsequent stages. The alignment of granularity ensures that all records in the dataset are represented at the same level of utility or metric. An example of utility is when the transaction date and time are both captured instead of only the date, if the date and time can be sourced for all inputs. Having more information (date and time) instead of less (date only) means the AI models can be accurate and effective at the specified tasks. An example for a metric is recording time in seconds instead of minutes, here again the higher granularity leads to better models. Attributes and data points should be checked ontologically, visually, and computationally for linear, cyclic, probabilistic, and differential relationships. If present, then domain expertise must be used to select the most effective representation of those data points or attributes. For instance, graph data must be handled using AI algorithms designed for cyclic relationships instead of linear models. The methods used for outlier detection will depend on the types of relationships, linear, cyclic, or probabilistic. Typically, outliers are removed from the dataset; however, depending on the problem description, outliers might be labeled and retained in the dataset to be used in model development or evaluation. Data quality primarily refers to the data being "fit for purpose." In its generic form, numerous frameworks, standards, and systems have been proposed for ensuring data quality.[55–58]

A further consideration of this stage is splitting the data for model development and evaluation. In principle, model evaluation is where the developed AI model is tested on a previously unseen scenario/data, and the result of this test is an indication of "intelligence" where the AI model has been able to generalize based on past experiences (training data). Data splitting typically takes two forms: the holdout method (or train-test split), where the dataset is split into two subsets, training and testing, typically as 80% training and 20% testing; secondly, the calibration method (or train-validate-test split), where the dataset is split three ways (the majority followed by two equal minorities, 60% training, 20% validation, and 20% testing, or 80% training, 10% validation, and 10% testing). Here, the validation phase is a calibration of the trained model where model parameters are fine-tuned using the validation split. N-fold cross-validation is an improvisation of the holdout method for smaller datasets where the number of records are limited. The original dataset is crafted into n instances by changing the 10% or 20% split of the testing subset; for instance, if $n = 7$ then the original dataset is split into $k = 7$ equal segments and for each nth instance of the dataset, the testing split will be a $k = 1, 2 \ldots 7$ segment. Using this approach, the entire dataset is utilized in an unbiased manner to evaluate the model n times.

External data acquisition

Data preparation and exploration stages together can expose limitations in the available data that makes it infeasible to build AI models. In such an instance, it is pertinent to investigate opportunities for external data acquisition. The acquisition of required data, in aggregate or detailed format from data brokers and data vendors, is a widely used short-term strategy. These brokers and vendors leverage public records, credit-scoring services, social media content, and third-party data sharing agreements to accumulate large volumes of data, which are then commercialized into aggregate products, such as sociodemographic or healthcare profiles. A formal risk assessment must be conducted before the acquisition of external data, focusing on the supply chain, pre-processing, practice of governance, and any other domain-specific policies of the data provider.

The ethical alignment as well as the sustainability of this approach are poorly formed. Therefore, it is recommended to develop a long-term data acquisition strategy based on trustworthy relationships with all stakeholders by being transparent on what data are collected and how they are used.

Data pre-processing

The data pre-processing stage ensures that all the data that have been acquired to build the AI model/application can be accurately input into the AI algorithm, with minimal compromise of accuracy, informational value, and data quality. This includes imputation, formatting, and transformation. Imputation is the identification of missing values and replacement with suitable substitutes. Imputation is generally attempted using statistical methods and some machine learning methods. In statistics, the simplest method is to use the mean, median, or mode of the available values; slightly more advanced methods include regression imputation where line-fitted values (without a residual) are used, or hot-deck imputation where replacements are randomly chosen from the same or a similar set of variables. Machine learning methods have also been adopted, such as the k-NN algorithm where the non-missing values of the neighbors are used for imputation, or more advanced libraries, such as DataWig,[59] which utilizes deep learning feature extractors. Data formatting is more straightforward where units of measurement, date format, or non-overlapping groups must be standardized across all variables in the dataset and the expected output of the AI model. Transformation is the conversion of non-numerical data types into numerical representations using methods such as label encoding or one-hot encoding. Each method has limitations that must also be checked and validated; for instance, one-hot encoding should not lead to multicollinearity in the dataset. It should be noted that data wrangling is a common term used to refer to the three stages up to pre-processing. This term was introduced by Terrizzano et al. in 2015,[60] and is equivalent to the three stages: data exploration, acquisition, and pre-processing.

Build initial AI model

This stage begins the development of an AI model by determining a suitable AI algorithm that represents the AI capabilities corresponding to the AI application. Figure 2 depicts a taxonomy for this mapping between application, capability, and algorithm. All current practical applications can be grouped into one or a combination of the four capabilities: prediction, classification, association, and optimization. When determining which algorithm should be used to build the model, the recommended approach is to start with "applications" and then follow through into "algorithms" via "capabilities." It is recommended practice to begin the model development process with a simple algorithm, default parameters, and default architecture. This enables a rigorous and validated approach that can also be interpreted,

CellPress
OPEN ACCESS

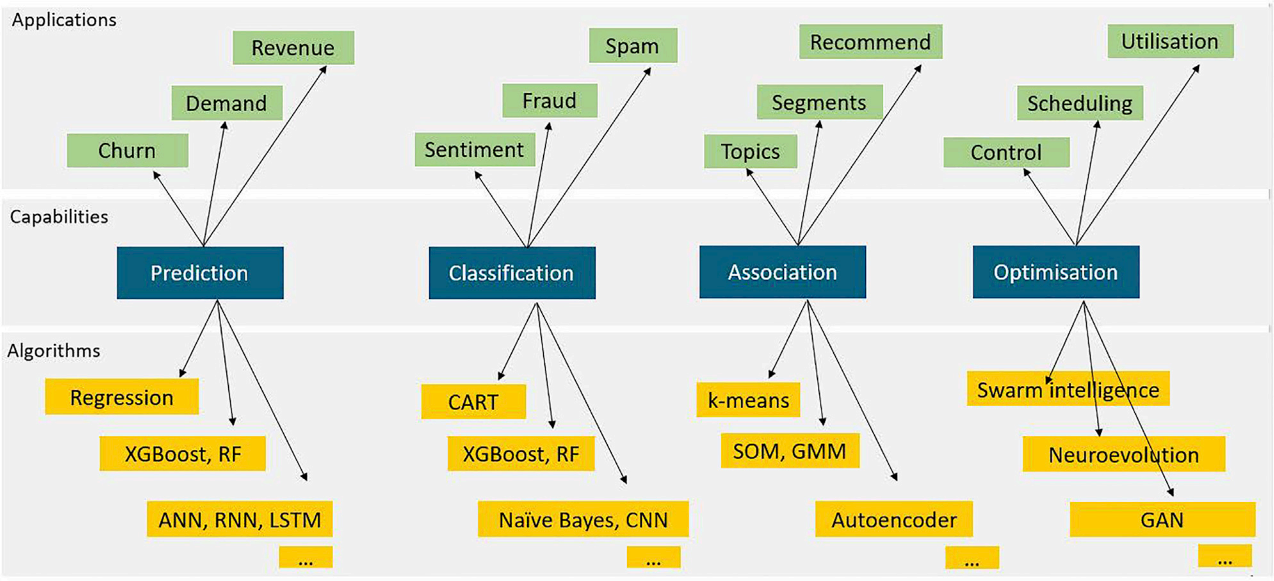

Figure 2. A taxonomy of AI algorithms, capabilities, and applications

presented, and documented. If this is a pre-trained model, then transfer learning or fine-tuning will be conducted at this stage, which follows the train-test phases or train-validate-test methods mentioned earlier. The model should be evaluated using the metrics designed for the type of AI capability and the algorithm itself. These are typically found in corresponding API documentation and worked examples. This initial model serves several purposes. Firstly, if the intended AI approach is feasible, based on the level of accuracy achieved using the default parameters. As noted in "developing a benchmark," the benchmark is typically a common-sense heuristic, such as human expertise in solving the same problem. Secondly, if it serves the purpose of validating the design and selection of AI capability as well as the datasets and attributes. Thirdly, the next steps and directions, typically in increasing complexity of algorithm and datasets.

Data augmentation

Informed by the output and evaluation of the initial model, data augmentation addresses limitations in the dataset that affect the model output. In contrast to data transformation, which is specific to variables, augmentation includes, class imbalance, feature engineering, and feature representation, the initial model will be re-run several times as the effectiveness of the augmentation measures are evaluated. Class imbalance is typically addressed using undersampling and oversampling techniques,[61,62] while feature engineering and feature representation are broad topics that span across distinct areas of study, such as signal processing techniques,[63,64] vector symbolic architectures,[65–67] and dynamic time warping.[68,69]

Develop a benchmark

As the first AI model is being developed, a suitable evaluation benchmark must be considered. This benchmark is typically drawn from a common-sense heuristic, such as human expertise in solving the same problem (for instance, in the anomaly detection) or the success rate of the human expert in detecting anomalies using the same input data. Alternatively, the benchmark can be drawn from the literature associated with the same algorithm or the industry sector. In addition to evaluating the model, the benchmark also serves the purpose of identifying what input vectors or attributes do not fit to the model, leading to the next stage of building multiple models or return to the data pre-processing stage if the data are an incomplete representation of the problem domain.

Build multiple AI models

The maturity of the AI discipline, mainly in terms of build technologies, such as Jupyter notebooks and the availability of feature-oriented algorithms as code libraries on repositories such as GitHub, have contributed toward the capability and time efficiency of building several AI models. Licensing for distribution and re-use specified in code repositories and any third-party content must be adhered to and documented to ensure that the developed application is legal and valid. Alternatively, the agility of modern programming languages (such as Python programming language) allows these algorithms to be re-implemented quickly without the constraints imposed by the code repository license. Reimplementation also ensures the integrity of the model, unlike prebuilt and pre-trained models, which link back to the preliminary risk assessment of the trustworthiness of the model and the source data.

As noted in the previous stage, the performance benchmark determines what is missing in the first model and, accordingly, the subsequent models would gradually increase model complexity. For instance, a classification problem can be first attempted using the fairly straightforward logistic regression, followed by the increasing complexity of decision tree, random forest, XGBoost, LightGBM, and deep neural network. The inverse of this approach has also been adopted and reported, where a more complex algorithm is used first and then regularization techniques are used to generalize the overfitted model. In both approaches, the dataset may not be representative, in which case the data pre-processing

stage must be revisited for exploration and evaluation of attributes and data points. When the required capability is not present in existing algorithms then new algorithms have to be developed based on novel research. This approach is only occasionally practiced when the state-of-the-art algorithms for a identified AI capability are inadequate at addressing the technical or computational challenges. Any new algorithm must be evaluated separately against state-of-the-art algorithms using benchmark datasets to establish utility and the differential value addition toward the AI capability. It is also recommended that new research yielding innovative outcomes are published and shared for the advancement of the AI discipline. Auditable autonomy is a recent innovation that can be leveraged when building multiple models, such as the use of brain-inspired neural computation principles and scalable deep learning architectures to design compact neural controllers.[70]

Evaluate primary metrics

The primary metrics build on the performance benchmark determined earlier. It is important to understand what the metric is representing and how it is being calculated, and numerous types of literature, from API documentation to case studies, contain this information. An effective evaluation metric should be accurate, robust, agnostic, scalable, and interpretable. Frequently used metrics are: accuracy, precision, recall, F1 score, root mean-squared error, purity, and entropy. The generalized formulation of a performance metric, $Outcome = model + error$, is useful to address the common misconception that "accuracy" is the only performance metric worth considering and selecting an appropriate metric when there are many options available. The evaluation metrics are also useful to compare all models across default and fine-tuned parameter settings, as well as to determine the bias variance trade-off.

AI model explainability

Model explainability (also known as model interpretability or explainable AI [XAI]) is a recent development driven by the ethical and regulatory need to make AI transparent. However, model development has also benefited from explainability as AI scientists are more informed on how the technicalities of attributes, the learning process, and model parameters contribute toward the expected AI outcome. AI transparency is required for complex models (such as gradient boosting or neural networks), where the flow of information from input vectors to decision output is obscured by many layers of distributed (or partial) computations. It is not possible to "unpack" or deconstruct this information flow, numerically or visually, in contrast to simpler models such as logistic regression or decision trees. Several survey articles have classified XAI methods using diverse dimensions, such as data-driven and knowledge-aware,[71] pixel-wise explanations,[72] intrinsic interpretability and post-hoc interpretability,[73] and model explanation, outcome explanation, model inspection, and transparent design.[74] In summary, XAI can be broadly categorized into intrinsic and extrinsic methods, where intrinsic methods are methods endemic to the algorithm that work alongside the AI capability, and extrinsic methods are typically post-processing techniques applied to the model output. Most AI algorithms have not been designed (or evolved) with intrinsic methods, therefore XAI is primarily a collection of extrinsic methods, such as partial dependence plots, individual conditional expectation, local interpretable model explanation, and Shapley addictive explanations (SHAP).

Evaluate secondary metrics

At this stage, the AI scientist hands over a prototypical and functional AI model to the AI/ML engineer. In addition to an effective demonstration of "intelligence" (at least one of the four AI capabilities) in performing the given task, the AI model must be computationally effective so that it can be deployed or operationalized for a broader audience. Therefore, a set of secondary metrics, computational (CPU) performance, memory performance, time-complexity, ethical implications, and convergence metrics are calculated in this stage. A further set of metrics focusing on the risk assesssment factors of privacy, cybersecurity, trust, robustness, explainability, interpretability, usability, and related social implications should also be formulated and evaluated. Emerging research on topics, such as adversarial robustness, will gradually become mainstay to address the risk of cyber threats and attacks on AI models. A standardized benchmark of adversarial robustness has been reported,[75] where it has been applied across 120+ models in image classification and made available for other application domains. The secondary metrics are not critical if the AI model is to be used by a smaller audience. Following the secondary metrics, a decision should be made on model compression, data hydration, and locality of deployment.

A set of metrics and tools that have manifested to address AI algorithmic bias, and ethical and legal challenges of AI, are known as the AI fairness metrics. As reported by Narayanan,[76] fairness does not pertain a consistent universally accepted definition, they demonstrate how at least 21 mathematical definitions of fairness lead to entirely different outcomes. Building fair AI models and removing AI bias is an active field of research with specialized terminology,[77] auditing predictive models,[78] bias detection and mitigation,[79] and counterfactual fairness.[80] AI technology providers have also worked toward transforming research into practice through the development of fairness libraries, such as AI Fairness 360 (AIF360) introduced by IBM,[81] the What-If Tool by Google Research,[82] and the OpenAI Gym by Open AI.[83] A robustness benchmark for adversarial attacks has also been proposed.[75]

AI model deployment and risk assessment

Also known as model serving, model scoring, and model production, in this stage, the evaluated model is deployed for operational use. It is smaller in scale to "operationalization," which is described next. Deployment would typically involve a smaller group of experts and users instead of organization-wide access. The primary considerations of deployment are real-time versus batch use of the AI model, the number of end-users and types of applications, the expected formats of output, the expected turnaround time, and the frequency of use.

Aligning with the preliminary risk assessment, a technical risk classification and analysis must be conducted in this stage as the AI model now integrates with external systems and processes when deployed. This stage also extends the initial deliberation of AI ethics, governance, and regulation into the deployment phase. The risks posed by the AI deployment should cover all stakeholders, oraganizational functions, government regulation, social norms, and any other societal implications following deployment. A risk register and risk assessment matrix can be used to further evaluate the criticality of each risk and document potential actions and mitigation strategies.

Post-deployment review

Depending on the industry sector and scope of project, an expert panel, steering committee, or regulatory body, will conduct a technical and ethics review of the entire project, from a datasets approach, AI model, to evaluate metrics and effectiveness. Compliance, standardization, post-implementation documentation, contracts, and service level agreements will also be administered in this stage. Specifically in healthcare, further investigations in the form of observational studies, small-scale clinical trials, training, and user-acceptance exercises will be conducted. In this stage, the team would also consider the legal protection of intellectual property in the form of patenting or the alternatives of trade secrecy that provides continuing protection (as patents expire) or defensive publications in academic journals that contribute toward the advancement of the discipline.

Operationalize using AI pipelines

Also known as AIOps or MLOps, this stage is an adaptation of the highly effective DevOps software automation capabilities into AI model deployment. An output of the Agile movement, Dyck et al.[84] formally defined DevOps as ‘‘a collaborative and multidisciplinary effort within an organization to automate continuous delivery of new software versions, while guaranteeing their correctness and reliability.’’ Several survey articles have reviewed the advantages, disadvantages, and challenges of DevOps[85,86] and its adaptation into AI.[87,88] In contrast to DevOps, AIOps is loosely defined given the dynamic nature of data received by an AI model and the output generated. Containers and microservices are utilized to develop the AI and data pipelines for operationalization. Data availability, collection, storage, pre-processing, versioning, and ethics are some of the main considerations for the data pipeline, while the AI pipeline should account for model compression, device compatibility, service definitions, versioning, auditing, re-training, maintenance, and monitoring. A microservice is an application with an atomic function. It has been more formally defined as an ‘‘independently deployable component of bounded scope that supports interoperability through message-based communication.’’[89] In AI, this will be the evaluated AI model that functions as a classifier or predictor. Although a microservice is standalone, it is more effectively executed inside a container or the containerization of that function. Containers are a cloud-based, platform as a service virtualization technology that bundles an application, its code, and dependencies with a computing platform for reusability, reliability, and time efficiency.[90] Containers enable technology-agnostic hyperautomation by abstracting diverse technologies to work together.

Hyperautomation of processes and systems

Hyperautomation is an emerging topic in industrial and knowledge work settings that aims to integrate and advance the efficiencies of automation with the productivity gains of AI. While the explicit goal of AI is to support/enable/augment human actions and decision making, hyperautomation attempts to takes this a step further by automating the actions and decision making within the boundaries of ethics and regulatory requirements. Most AI projects conclude when the human effort has been enriched by the AI model/s, but an increasing trend in innovative AI solutions is to push toward hyperautomation.

In knowledge work, automation takes the form of robotic process automation (RPA),[91,92] and, in industrial settings, it manifests as cyber-physical systems and intelligent robotics.[93,94] As part of the AI life cycle, an operationalized AI service will be interconnected with a process automation pipeline to deliver hyperautomated processes and systems. The capabilities of the AI solution must be demonstrated to downstream and upstream process owners and stakeholders to generate interest toward a pilot phase of hyperautomation. Following this pilot phase, the performance gains in efficiency, effectiveness, and productivity must be measured and evaluated against previous configurations to develop a business case for deployment at scale.

A further consideration in this stage is the ‘‘system of systems engineering view of the deployed and hyperautomated AI model.’’ As deliberated in the systems and software engineering practice ISO/IEC/IEEE 15288:2015, the challenges in this respect are resources, technical specification, verification and validation process, software system execution environment, configuration and change management processes, re-use, standardization, education, and training.[95]

Monitor and evaluate performance

The AI model that has either been deployed independently or integrated as a hyperautomation process will be monitored and evaluated in this final stage of the life cycle. The main evaluation criteria are representative of the technology itself, diverse individuals in diverse settings utilizing the technology and value generated by the technology. The technology is evaluated using model drift and model staleness; end-user activity measures the people criterion, and return on investment (ROI) measures value generation. Model drift denotes decreasing accuracy of the model due to the changing nature of the data, which can be addressed by retraining the model with more recently accumulated data points that capture these changes or drifts. On the other hand, model staleness is caused by changes in the problem or environment description underlying the model design and development. Addressing staleness requires a fundamental rethink of the model architecture, inputs, algorithm, and parameters. A stale model will trigger a new iteration of the full life cycle. Continuous monitoring of end-user activity is critical to find out if and how the model is contributing toward organizational functions. The level of end-user activity depends on each use case and metrics can be drawn from adoption, questions, frequency of use, use/revision of documentation, feedback, and requests for features. Finally, although ROI is not directly visible like most other knowledge work, it can be determined using several types of metrics, such as reduced costs (due to reductions in turnaround time, human effort, human skill), increased revenue (due to new revenue streams, customer satisfaction, increased market share), and productivity gains (such as reduced errors, low employee turnover, increased agility of teams, and workflows).

These 19 stages provide comprehensive coverage of the design, development, and deployment of an AI solution. In Tables S1 and S2, we provide further insight into the life cycle through its exemplification in two distinct application areas: conception to production of a micro-profiling service for a large energy retailer (Table S1), and conception to production of an AI conversational agent (chatbot) for patient-centered healthcare (Table S2).

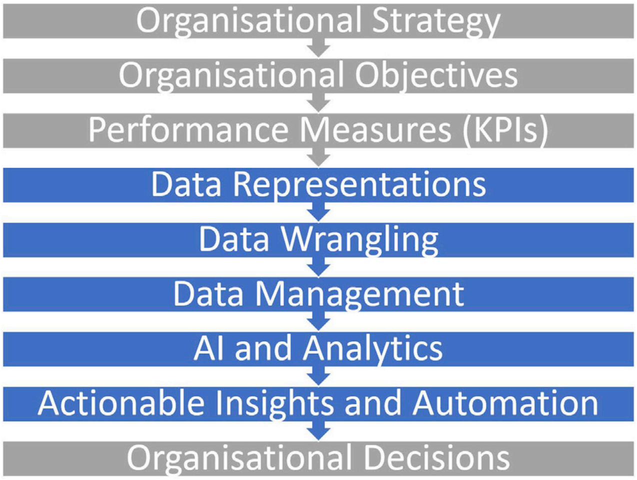

Figure 3. The organizational context of AI

An AI taxonomy

In both research and industry engagement, we have observed how the depth and breadth of the AI discipline can inhibit the functional or practical adoption of its primary capabilities. Drawing on these observations, we have formulated a technical and organizational AI taxonomy that can aid in improved awareness, positioning, adoption, and value creation from AI. Figure 2 depicts the technical AI taxonomy, composed of three layers: AI applications, AI capabilities, and AI algorithms. The top layer, AI applications, is typically determined from the problem/environment description. Although numerous, these applications can be condensed into one of four capabilities, prediction, classification, association, and optimization, or a combination of these. The bottom layer represents AI algorithms, which again are numerous and overlapping. As the intermediate layer, AI capabilities provide clarity and enable navigation from AI applications to AI algorithms. The four AI capabilities can be further deliberated as follows: prediction represents the numerical prediction of continuous, discrete, time series, and sequential data; classification represents categorical predictions, such as categorization, object detection, anomaly detection, concept detection, and sentiment analysis; association represents intelligence from unlabeled data, such as clustering, association rule mining, feature selection, encoding, and dimensionality reduction; and finally, optimization represents the generation of improved solutions for scheduling, planning, control, generation, and simulation type problems. Figure 3 presents an organization taxonomy that positions the AI life cycle and AI capabilities within the flow of activities and information between organizational strategy to decision making. The 19 stages have been condensed into five technical functions (in blue) to align with the organizational functions, depicted in gray.

DISCUSSION

Conclusion

In this article, we have presented the CDAC AI life cycle for the design, development, and deployment of AI systems and solutions, preceded by a preliminary risk assessment for the adoption of AI. The life cycle consists of three phases (design, develop, and deploy) and 19 constituent stages across the three phases from conception to production. The life cycle addresses several key limitations in related methods and approaches, exclusive focus on AI, depth of technical detail of each phase, responsibilities of an AI team, and the contribution of pre-trained models, code repositories, and ethics and governance frameworks toward expediting AI project outcomes and enhancing inclusivity.

As limitations of this study, we note that the generic nature of the life cycle may not be applicable to certain domains of application with specific inclusion criteria or regulatory requirements. This also applies to the preliminary risk assessment, which will include other risk factors influenced by new research and technological advancements. A further limitation is the expansive disposition of AI to include data analytics and data science projects that would utilize a combination of techniques in statistics, data modeling, and natural language processing, and also leverage prebuilt tools and functions of convenience on cloud platforms, data lakes, and analytics dashboards. We reiterate that a subset of the 19 stages would still be applicable for such diverse initiatives but also appreciate that refined approaches can be proposed as future work.

We anticipate that the CDAC AI life cycle and the preliminary risk assessment will contribute toward an informed practice of AI, as well as the increased awareness, knowledge, and transparency of AI and its capabilities. The technical and organizational AI taxonomies will contribute toward nuanced engagement between AI scientists, AI engineers, and other stakeholders during the three phases of the life cycle. The organizational context will further integrate the AI team and their solutions with other stakeholders, such as senior management and the executive, in working toward an inclusive organizational strategy. In conclusion, the CDAC AI life cycle advances effective, ethical, and inclusive research applications, practice of AI, and policy development, that advance AI for the good of humanity.

EXPERIMENTAL PROCEDURES

Resource availability

Lead contact

Requests for further information can be directed to the lead contact Daswin De Silva at d.desilva@latrobe.edu.au.

Materials availability

This study did not generate new unique reagents.

Data and code availability

This study did not generate new data or code.

SUPPLEMENTAL INFORMATION

Supplemental information can be found online at https://doi.org/10.1016/j.patter.2022.100489.

ACKNOWLEDGMENTS

The authors acknowledge the support and commitment of the CDAC team toward the advancement AI innovations that initiated and enabled this body of work. We also acknowledge and appreciate all feedback and comments provided by the editor and the panel of anonymous reviewers. Your feedback has significantly improved the quality and contributions of this article.

AUTHOR CONTRIBUTIONS

Conceptualization, D.D.S. and D.A.; methodology, D.D.S. and D.A.; investigation, D.D.S.; writing – original draft, D.D.S.; writing – review & editing, D.D.S. and D.A.

DECLARATION OF INTERESTS

The authors declare no competing interests.

Received: November 15, 2021
Revised: March 5, 2022
Accepted: March 16, 2022
Published: April 13, 2022

REFERENCES

1. Partridge, D. (1986). Engineering artificial intelligence software. Artif. Intell. Rev. *1*, 27–41.
2. Shearer, C. (2000). The crisp-dm model: the new blueprint for data mining. J. Data Warehous. *5*, 13–22.
3. Rollins, J. (2015). Foundational Methodology for Data Science (Whitepaper: Domino Data Lab, Inc.).
4. Moyle, S., and Jorge, A. (2001). Ramsys-a methodology for supporting rapid remote collaborative data mining projects. ECML/PKDD01 Workshop: Integrating Aspects of Data Mining, Decision Support and Meta-Learning (IDDM-2001), *64*.
5. Martinez-Plumed, F., Contreras-Ochando, L., Ferri, C., Orallo, J.H., Kull, M., Lachiche, N., Quintana, M.J.R., and Flach, P.A. (2019). Crisp-dm Twenty Years Later: From Data Mining Processes to Data Science Trajectories (IEEE Transactions on Knowledge and Data Engineering).
6. Cios, K.J., and Kurgan, L.A. (2005). Trends in data mining and knowledge discovery. In Advanced techniques in knowledge discovery and data mining (Springer), pp. 1–26.
7. Wu, X., Zhu, X., Wu, G.-Q., and Ding, W. (2013). Data mining with big data. IEEE Trans. Knowl. Data Eng. *26*, 97–107.
8. Foroughi, F., and Luksch, P. (2018). Data science methodology for cybersecurity projects. Preprint at arXiv. 1803.04219.
9. Haakman, M., Cruz, L., Huijgens, H., and van Deursen, A. (2021). Ai lifecycle models need to be revised. Empir. Software Eng. *26*, 1–29.
10. Ericson, G., Rohm, W.A., Martens, J., Sharkey, K., Casey, C., Harvey, B., and Schonning, N. (2017). Team Data Science Process Documentation, *11*, p. 2019.
11. Amershi, S., Begel, A., Bird, C., DeLine, R., Gall, H., Kamar, E., Nagappan, N., Nushi, B., and Zimmermann, T. (2019). Software engineering for machine learning: a case study. In 2019 IEEE/ACM 41st International Conference on Software Engineering: Software Engineering in Practice (ICSE-SEIP) (IEEE), pp. 291–300.
12. "Bunji - a good friend to chat." https://play.google.com/store/apps/details?id=au.edu.latrobe.cdac.bunji. Accessed: 01-08-2021.
13. Haputhanthri, D., De Silva, D., Sierla, S., Alahakoon, D., Nawaratne, R., Jennings, A., and Vyatkin, V. (2021). Solar irradiance nowcasting for virtual power plants using multimodal long short-term memory networks. Front. Energy Res. *9*, 469.
14. Kahawala, S., De Silva, D., Sierla, S., Alahakoon, D., Nawaratne, R., Osipov, E., Jennings, A., and Vyatkin, V. (2021). Robust multi-step predictor for electricity markets with real-time pricing. Energies *14*, 4378.
15. Osipov, E., Kahawala, S., Haputhanthri, D., Kempitiya, T., De Silva, D., Alahakoon, D., and Kleyko, D. (2021). Hyperseed: unsupervised learning with vector symbolic architectures. Preprint at arXiv. 2110.08343.
16. Adikari, A., Nawaratne, R., De Silva, D., Ranasinghe, S., Alahakoon, O., Alahakoon, D., et al. (2021). Emotions of covid-19: content analysis of self-reported information using artificial intelligence. J. Med. Internet Res. *23*, e27341.
17. Adikari, A., de Silva, D., Ranasinghe, W.K., Bandaragoda, T., Alahakoon, O., Persad, R., Lawrentschuk, N., Alahakoon, D., and Bolton, D. (2020). Can online support groups address psychological morbidity of cancer patients? an artificial intelligence based investigation of prostate cancer trajectories. PLoS ONE *15*, e0229361.
18. De Silva, D., Ranasinghe, W., Bandaragoda, T., Adikari, A., Mills, N., Iddamalgoda, L., Alahakoon, D., Lawrentschuk, N., Persad, R., Osipov, E., et al. (2018). Machine learning to support social media empowered patients in cancer care and cancer treatment decisions. PLoS ONE *13*, e0205855.
19. Alahakoon, D., Nawaratne, R., Xu, Y., De Silva, D., Sivarajah, U., and Gupta, B. (2020). Self-building artificial intelligence and machine learning to empower big data analytics in smart cities. Inf. Syst. Front. 1–20.
20. Nallaperuma, D., De Silva, D., Alahakoon, D., and Yu, X. (2018). Intelligent detection of driver behavior changes for effective coordination between autonomous and human driven vehicles. In IECON 2018-44th Annual Conference of the IEEE Industrial Electronics Society (IEEE), pp. 3120–3125.
21. Nallaperuma, D., Nawaratne, R., Bandaragoda, T., Adikari, A., Nguyen, S., Kempitiya, T., De Silva, D., Alahakoon, D., and Pothuhera, D. (2019). Online incremental machine learning platform for big data-driven smart traffic management. IEEE Trans. Intell. Transport. Syst. *20*, 4679–4690.
22. De Silva, D., Sierla, S., Alahakoon, D., Osipov, E., Yu, X., and Vyatkin, V. (2020). Toward intelligent industrial informatics: a review of current developments and future directions of artificial intelligence in industrial applications. IEEE Ind. Elect. Mag. *14*, 57–72.
23. Berghoff, C., Neu, M., and von Twickel, A. (2020). Vulnerabilities of connectionist ai applications: evaluation and defense. Front. Big Data *3*, 23.
24. Berghoff, C., Bielik, P., Neu, M., Tsankov, P., and Twickel, A.v. (2021). Robustness testing of ai systems: a case study for traffic sign recognition. In IFIP International Conference on Artificial Intelligence Applications and Innovations (Springer), pp. 256–267.
25. Taeihagh, A. (2021). Governance of artificial intelligence. Pol. Soc. *40*, 137–157.
26. Agrawal, V. (2017). A comparative study on information security risk analysis methods. J. Comput. *12*, 57–67.
27. Goel, R., Kumar, A., and Haddow, J. (2020). Prism: A Strategic Decision Framework for Cybersecurity Risk Assessment (Information & Computer Security).
28. Garvey, P.R., and Lansdowne, Z.F. (1998). Risk matrix: an approach for identifying, assessing, and ranking program risks. Air Force J. Logist. *22*, 18–21.
29. Završnik, A. (2021). Algorithmic justice: algorithms and big data in criminal justice settings. Eur. J. Criminol. *18*, 623–642.
30. Marjanovic, O., Cecez-Kecmanovic, D., and Vidgen, R. (2021). Theorising algorithmic justice. Eur. J. Inf. Syst. 1–19.
31. Muller, M., and Liao, Q.V. (2017). Exploring Ai Ethics and Values through Participatory Design Fictions (Human Computer Interaction Consortium).
32. Bratteteig, T., and Verne, G. (2018). Does ai make pd obsolete? exploring challenges from artificial intelligence to participatory design. In Proceedings of the 15th Participatory Design Conference: Short Papers, Situated Actions, Workshops and Tutorial, *2*, pp. 1–5.
33. Mittelstadt, B. (2019). Principles alone cannot guarantee ethical ai. Nat. Mach. Intell. *1*, 501–507.
34. IEEE (2018). The IEEE Global Initiative on Ethics of Autonomous and Intelligent Systems. Ethically Aligned Design, 2 ed. (IEEE).
35. European-Commission (2019). AI Ethics Guidelines by the High-Level Expert Group on Artificial Intelligence, 1 ed. (EU Commission).
36. European-Commission (2021). Proposal for a Regulation of the European Parliament and of the Council Laying Down Harmonised Rules on Artificial Intelligence (Artificial Intelligence Act) and Amending Certain Union Legislative Acts.

37. Krizhevsky, A., Sutskever, I., and Hinton, G.E. (2012). Imagenet classification with deep convolutional neural networks. Adv. Neural Inf. Process. Syst. *25*, 1097–1105.
38. He, K., Zhang, X., Ren, S., and Sun, J. (2016). Deep residual learning for image recognition. In Proceedings of the IEEE conference on computer vision and pattern recognition, pp. 770–778.
39. Devlin, J., Chang, M.-W., Lee, K., and Toutanova, K. (2018). Bert: pre-training of deep bidirectional transformers for language understanding. Preprint at arXiv. 1810.04805.
40. Radford, A., Narasimhan, K., Salimans, T., and Sutskever, I. (2018). Improving language understanding by generative pre-training. Preprint at arXiv. cs.ubc.ca.
41. Eckart de Castilho, R., Dore, G., Margoni, T., Labropoulou, P., and Gurevych, I. (2018). A Legal Perspective on Training Models for Natural Language Processing (Enlighten: Publications).
42. Eshraghian, J.K. (2020). Human ownership of artificial creativity. Nat. Mach. Intell. *2*, 157–160.
43. Kimball, R., and Ross, M. (2011). The Data Warehouse Toolkit: The Complete Guide to Dimensional Modeling (John Wiley & Sons).
44. Inmon, W.H. (1996). The data warehouse and data mining. Commun. ACM *39*, 49–51.
45. O'Leary, D.E. (2014). Embedding ai and crowdsourcing in the big data lake. IEEE Intell. Syst. *29*, 70–73.
46. Halevy, A.Y., Korn, F., Noy, N.F., Olston, C., Polyzotis, N., Roy, S., and Whang, S.E. (2016). Managing google's data lake: an overview of the goods system. IEEE Data Eng. Bull. *39*, 5–14.
47. Gorelik, A. (2019). The Enterprise Big Data Lake: Delivering the Promise of Big Data and Data Science (O'Reilly Media).
48. Li, T., Sahu, A.K., Talwalkar, A., and Smith, V. (2020). Federated learning: challenges, methods, and future directions. IEEE Signal. Process. Mag. *37*, 50–60.
49. Bonawitz, K., Eichner, H., Grieskamp, W., Huba, D., Ingerman, A., Ivanov, V., Kiddon, C., Konečnỳ, J., Mazzocchi, S., McMahan, H.B., et al. (2019). Towards federated learning at scale: system design. Preprint at arXiv. 1902.01046.
50. Bengio, Y., Courville, A., and Vincent, P. (2013). Representation learning: a review and new perspectives. IEEE Trans. Pattern Anal. Mach. Intell. *35*, 1798–1828.
51. Hamilton, W.L., Ying, R., and Leskovec, J. (2017). Representation learning on graphs: methods and applications. Preprint at arXiv. 1709.05584.
52. Wang, M.Z., and Hayden, B.Y. (2021). Latent learning, cognitive maps, and curiosity. Curr. Opin. Behav. Sci. *38*, 1–7.
53. Yoo, S.B.M., Hayden, B.Y., and Pearson, J.M. (2021). Continuous decisions. Philos. Trans. R. Soc. B *376*, 20190664.
54. "2018 Reform of Eu Data Protection Rules."
55. Sidi, F., Panahy, P.H.S., Affendey, L.S., Jabar, M.A., Ibrahim, H., and Mustapha, A. (2012). Data quality: a survey of data quality dimensions. In 2012 International Conference on Information Retrieval & Knowledge Management (IEEE), pp. 300–304.
[56]. Cai, L., and Zhu, Y. (2015). The challenges of data quality and data quality assessment in the big data era. Data Sci. J. *14*, 1–10.
57. Gupta, N., Patel, H., Afzal, S., Panwar, N., Mittal, R.S., Guttula, S., Jain, A., Nagalapatti, L., Mehta, S., Hans, S., et al. (2021). Data quality toolkit: automatic assessment of data quality and remediation for machine learning datasets. Preprint at arXiv. 2108.05935.
58. Gupta, N., Mujumdar, S., Patel, H., Masuda, S., Panwar, N., Bandyopadhyay, S., Mehta, S., Guttula, S., Afzal, S., Sharma Mittal, R., et al. (2021). Data quality for machine learning tasks. In Proceedings of the 27th ACM SIGKDD Conference on Knowledge Discovery & Data Mining, pp. 4040–4041.
59. Biessmann, F., Rukat, T., Schmidt, P., Naidu, P., Schelter, S., Taptunov, A., Lange, D., and Salinas, D. (2019). Datawig: missing value imputation for tables. J. Mach. Learn. Res. *20*, 175–181.
60. Terrizzano, I.G., Schwarz, P.M., Roth, M., and Colino, J.E. (2015). Data Wrangling: The Challenging Yourney from the Wild to the Lake (CIDR).
61. Yap, B.W., Abd Rani, K., Abd Rahman, H.A., Fong, S., Khairudin, Z., and Abdullah, N.N. (2014). An application of oversampling, undersampling, bagging and boosting in handling imbalanced datasets. In Proceedings of the first international conference on advanced data and information engineering (DaEng-2013) (Springer), pp. 13–22.
62. Mohammed, R., Rawashdeh, J., and Abdullah, M. (2020). Machine learning with oversampling and undersampling techniques: overview study and experimental results. In 2020 11th International Conference on Information and Communication Systems (ICICS) (IEEE), pp. 243–248.
63. Kassam, S.A., and Poor, H.V. (1985). Robust techniques for signal processing: a survey. Proc. IEEE *73*, 433–481.
64. Yu, N., Li, Z., and Yu, Z. (2018). Survey on encoding schemes for genomic data representation and feature learning—from signal processing to machine learning. Big Data Mining and Analytics *1*, 191–210.
65. Kanerva, P. (2009). Hyperdimensional computing: an introduction to computing in distributed representation with high-dimensional random vectors. Cogn. Comput. *1*, 139–159.
66. Gayler, R.W. (2004). Vector symbolic architectures answer jackendoff's challenges for cognitive neuroscience. Preprint at arXiv. cs/0412059.
67. Kleyko, D. (2016). Pattern Recognition with Vector Symbolic architectures, PhD Thesis (Luleå tekniska universitet).
68. Keogh, E.J., and Pazzani, M.J. (2000). Scaling up dynamic time warping for datamining applications. In Proceedings of the sixth ACM SIGKDD international conference on Knowledge discovery and data mining, pp. 285–289.
69. Li, H., Liu, J., Yang, Z., Liu, R.W., Wu, K., and Wan, Y. (2020). Adaptively Constrained Dynamic Time Warping for Time Series Classification and Clustering, *534* (Information Sciences), pp. 97–116.
70. Lechner, M., Hasani, R., Amini, A., Henzinger, T.A., Rus, D., and Grosu, R. (2020). Neural circuit policies enabling auditable autonomy. Nat. Mach. Intell. *2*, 642–652.
71. Li, X.-H., Cao, C.C., Shi, Y., Bai, W., Gao, H., Qiu, L., Wang, C., Gao, Y., Zhang, S., Xue, X., et al. (2020). A Survey of Data-Driven and Knowledge-Aware Explainable Ai (IEEE Transactions on Knowledge and Data Engineering).
72. Bach, S., Binder, A., Montavon, G., Klauschen, F., Müller, K.-R., and Samek, W. (2015). On pixel-wise explanations for non-linear classifier decisions by layer-wise relevance propagation. PLoS ONE *10*, e0130140.
73. Du, M., Liu, N., and Hu, X. (2019). Techniques for interpretable machine learning. Commun. ACM *63*, 68–77.
74. Guidotti, R., Monreale, A., Ruggieri, S., Turini, F., Giannotti, F., and Pedreschi, D. (2018). A survey of methods for explaining black box models. ACM Comput. Surv. *51*, 1–42.
75. Croce, F., Andriushchenko, M., Sehwag, V., Debenedetti, E., Flammarion, N., Chiang, M., Mittal, P., and Hein, M. (2020). Robustbench: a standardized adversarial robustness benchmark. Preprint at arXiv. 2010.09670.
76. Narayanan, A. (2018). Translation tutorial: 21 fairness definitions and their politics. Proc. Conf. Fairness Accountability Transp., *1170* (New York).
77. Bellamy, R.K., Dey, K., Hind, M., Hoffman, S.C., Houde, S., Kannan, K., Lohia, P., Martino, J., Mehta, S., Mojsilović, A., et al. (2019). Ai fairness 360: an extensible toolkit for detecting and mitigating algorithmic bias. IBM J. Res. Dev. *63*, 1–4.
78. Adebayo, J.A. (2016). FairML: ToolBox for Diagnosing Bias in Predictive modeling, PhD Thesis (Massachusetts Institute of Technology).
79. Friedler, S.A., Scheidegger, C., Venkatasubramanian, S., Choudhary, S., Hamilton, E.P., and Roth, D. (2019). A comparative study of fairness-enhancing interventions in machine learning. In Proceedings of the conference on fairness, accountability, and transparency, pp. 329–338.
80. Kusner, M.J., Loftus, J.R., Russell, C., and Silva, R. (2017). Counterfactual fairness. Preprint at arXiv. 1703.06856.

81. Bellamy, R.K., Dey, K., Hind, M., Hoffman, S.C., Houde, S., Kannan, K., Lohia, P., Martino, J., Mehta, S., Mojsilovic, A., et al. (2018). Ai fairness 360: an extensible toolkit for detecting, understanding, and mitigating unwanted algorithmic bias. Preprint at arXiv. 1810.01943.
82. Wexler, J., Pushkarna, M., Bolukbasi, T., Wattenberg, M., Viégas, F., and Wilson, J. (2019). The what-if tool: interactive probing of machine learning models. IEEE Trans. Visual. Comput. Graph. *26*, 56–65.
83. Brockman, G., Cheung, V., Pettersson, L., Schneider, J., Schulman, J., Tang, J., and Zaremba, W. (2016). Openai gym. Preprint at arXiv. 1606.01540.
84. Dyck, A., Penners, R., and Lichter, H. (2015). Towards definitions for release engineering and devops. In 2015 IEEE/ACM 3rd International Workshop on Release Engineering (IEEE), p. 3.
85. Ebert, C., Gallardo, G., Hernantes, J., and Serrano, N. (2016). Devops. IEEE Softw. *33*, 94–100.
86. Leite, L., Rocha, C., Kon, F., Milojicic, D., and Meirelles, P. (2019). A survey of devops concepts and challenges. ACM Comput. Surv. *52*, 1–35.
87. Mäkinen, S., Skogström, H., Laaksonen, E., and Mikkonen, T. (2021). Who needs mlops: what data scientists seek to accomplish and how can mlops help?. Preprint at arXiv. 2103.08942.
88. Treveil, M., Omont, N., Stenac, C., Lefevre, K., Phan, D., Zentici, J., Lavoillotte, A., Miyazaki, M., and Heidmann, L. (2020). Introducing MLOps (O'Reilly Media, Inc.).
89. Nadareishvili, I., Mitra, R., McLarty, M., and Amundsen, M. (2016). Microservice Architecture: Aligning Principles, Practices, and Culture (O'Reilly Media, Inc.).
90. Pahl, C., Brogi, A., Soldani, J., and Jamshidi, P. (2017). Cloud container technologies: a state-of-the-art review. IEEE Trans. Cloud Comput. *7*, 677–692.
91. Van der Aalst, W.M., Bichler, M., and Heinzl, A. (2018). Robotic Process Automation.
92. Syed, R., Suriadi, S., Adams, M., Bandara, W., Leemans, S.J., Ouyang, C., ter Hofstede, A.H., van de Weerd, I., Wynn, M.T., and Reijers, H.A. (2020). Robotic process automation: contemporary themes and challenges. Comput. Ind. *115*, 103162.
93. Rajkumar, R., Lee, I., Sha, L., and Stankovic, J. (2010). Cyber-physical systems: the next computing revolution. In Design automation conference (IEEE), pp. 731–736.
94. Xu, L.D., and Duan, L. (2019). Big data for cyber physical systems in industry 4.0: a survey. Enterprise Inf. Syst. *13*, 148–169.
95. Alvarez-Rodríguez, J.M., Zuñiga, R.M., Pelayo, V.M., and Llorens, J. (2019). Challenges and opportunities in the integration of the systems engineering process and the ai/ml model lifecycle. In INCOSE International Symposium, *29* (Wiley Online Library), pp. 560–575.

Patterns, Volume *3*

Supplemental information

An artificial intelligence life cycle:

From conception to production

Daswin De Silva and Damminda Alahakoon

Table S1: Application of the CDAC AI Lifecycle for Conception to Production of a Micro-Profiling Service for a Large Energy Retailer

**Stages of the AI Lifecycle**	**Conception to production of a micro-profiling service for a large energy retailer**
Identify and formulate the problem	Energy consumption based on lifestyle and generation using rooftop solar and battery storage has created a diversity of energy consumers that cannot be classified into simple tariffs and plans.<br>How can an AI be used to identify micro-profiles that encapsulate energy usage patterns, generation patterns and lifestyle factors influencing both aspects so that a more comprehensive and inclusive tariff structure can be developed?
Review data and AI ethics	Preserve privacy and confidentiality of energy consumers and the data they have shared with the retailer. Ensure identifying customer information is excluded from the final micro profiles generated. Seek ethics approval from the organisational ethics committee or submit a report to the regulatory body for the sector.
Review technical literature on AI algorithms, applications and pre-trained models	Review and evaluate all segmentation and clustering algorithms, specifically focusing on those applied in customer profiling applications. Consider the use of pre-trained models for demographics and geographic profiling
Data preparation	Conduct a data/ database audit of all customer information recorded within the organisation, such as demographic, behavioural, psychometric, transactions, consultations, feedback data.
Data exploration	Evaluate the data in terms of attributes, records, data quality measures and relevance to the expected solution. Use visualisations, statistical techniques, correlation analysis and sampling methods.
External data acquisition	In consultation with domain experts assess the quality and quantity of data that they would be using to profile customers for other intentions, such as sales, marketing or customer relationship management. Explore options for collecting more nuanced demographic and lifestyle information about the target segments serviced by the retailer.
Data pre-processing	Apply relevant pre-processing techniques such as missing value imputation, range/format checks, transformation of categorical and textual data into numerical format that contain the appropriate semantics for the task at hand
Build initial AI model	Start with the least complex segmentation / clustering algorithm that can accommodate the volume of data that has been collected for this exercise. Determine a number of levels of profiles to be generated, define what is meant by 'micro' for this segmentation algorithm.
Data augmentation	Consider the use of externally sourced data or integrated/ fused data sequences such as consumption and generation patterns combined, or lifestyle factors and suburb profiles fused together.
Develop a benchmark	The current profiling scheme can be directly used as a benchmark to evaluate the micro profiles, or the profiles recommended by a human expert can also be used.
Build multiple AI models	Build new models in increasing algorithmic complexity, also consider including all data dimensions or combinations of dimensions

Evaluate primary metrics	Starting with the standard metrics, accuracy, precision, recall and F1 score, consider developing metrics that incorporate domain intricacies
AI model explainability	Utilise XAI methods and libraries to draw meaning and causality from the generated micro profiles, starting with the attribute densities and developing into combinatorial factors.
Evaluate secondary metrics	Computational performance and use of memory in re-segmentation and also mapping new data into one or more micro profiles.
AI model deployment and risk assessment	Deploy the model on a suitable infrastructure specification that supports multiple end-users and use cases.<br>Determine the risks of misclassification, noise, excess segmentation and low value segments that do not contribute to tariff design
Post-deployment review	Compliance, standardisation, post-implementation documentation, as well as service level agreements for model use and maintenance
Operationalise using AI pipelines	Utilise a microservices platform to scale up the deployment of profiles to service different use cases
Hyperautomation of processes and systems	Link up the profiles to automated services such as billing, invoicing, customer experience, planning and budgeting
Monitor and evaluate performance	Monitor performance metrics for all use cases when deployed and continuous evaluation to suit evolving needs and expectations.

Table S2: Application of the CDAC AI Lifecycle for Conception to Production of an AI Conversational Agent for Patient-Centred Healthcare

Stages of the AI Lifecycle	Conception to production of an AI conversational agent (chatbot) for patient-centred healthcare
Identify and formulate the problem	Increasing smartphone ownership and widespread use of the Internet to find health-related information has created an organisational void where individuals prefer unverified online resources over formal healthcare services for informational support and emotional support.<br><br>How can an AI chatbot be used to address this organisational void by providing personalised, intuitive, patient-centred support and information that is structured and approved by healthcare practitioners?
Review data and AI ethics	Healthcare regulation and medical ethics ensure privacy and confidentiality of patient and clinical data, with access being limited to approved individuals, applications and activities.<br><br>When investigating the potential of AI for addressing this organisational void, AI, data, patient ethics approval must be sought from an appropriate body and a representative terms and conditions must be presented to the end-user before they engage with the AI chatbot. The preference should be towards complete anonymity where no identifying information is collected by the AI service.
Review technical literature on AI algorithms, applications and pre-trained models	Review and evaluate proprietary and open-source platforms for building out a chatbot, such as Google Dialogflow, Azure bot service, Rasa. Review and evaluate pre-trained language models that can be plugged into a conversational service, as well as suitable prediction, classification and segmentation algorithms.
Data preparation	Conduct a data/ database audit of all conversational records available within the organisational systems. Determine the data structures and models used and attempt to integrate these into a unified structure that is fit for purpose.
Data exploration	Evaluate the data in terms of attributes, records, data quality measures and relevance to the expected solution. Use visualisations, statistical techniques and sampling methods.
External data acquisition	In consultation with domain experts (healthcare practitioners), assess the quality and quantity of conversational records for building out an intelligent chatbot service. If insufficient explore third party datasets provided by the chatbot platform providers and other research groups or organisations working in this space.
Data pre-processing	Apply relevant natural language pre-processing techniques (such as abbreviated terms, medical terms, emojis, short form sentences) that

	will structure and normalise the datasets to suit the conversation training process.
Build initial AI model	Explore several configurations for the AI capabilities of a chatbot, such as classification of end-user inputs into tasks or intents, prediction of the next question or comment by the end-user, using segmentation output to determine what type of end-user and what kind of health services might be required (given that the end-user is anonymous and no exchange of identifying data), optimise the recommended self-help or healthcare advice provided in terms of the emotional or informational needs expressed.
Data augmentation	For the four AI capabilities, assess the effectiveness of all the initial models and then explore opportunities for augmentation such as, more training data for selected parts of a conversation, dynamic time warping to align intents and actions required from the chatbot
Develop a benchmark	Quantify the level of accuracy expected from a human operator, along the scale of a beginner to an expert. i.e. know how many questions or comments will be misunderstood in the first round, second attempt and third.
Build multiple AI models	Explore more configurations of the chatbot information flow, also varying the algorithms and parameters used for each sub task
Evaluate primary metrics	Starting with the standard metrics, accuracy, precision, recall and F1 score, consider developing metrics that incorporate domain intricacies
AI model explainability	Develop the capacity to explain and interpret as a sequence of words or phrases why and how a certain response was generated.
Evaluate secondary metrics	Computational performance and use of memory for conversations of varying length and depth, taking into account repeat questions and follow-up clarification questions
AI model deployment and risk assessment	Deploy the model on a suitable infrastructure specification that supports multiple end-users without any interruptions or performance issues.<br><br>Determine the risks of drop-outs, incorrect responses, turnaround time for direct human involvement as well as the boundaries of conversation.
Post-deployment review	Compliance, standardisation, post-implementation documentation, and small-scale clinical trials of the effectiveness of the chatbot in diverse healthcare settings
Operationalise using AI pipelines	Utilise a microservices platform to scale up the deployment of the chatbot to serve many individuals across multiple healthcare functions

Hyperautomation of processes and systems	Link up the chatbot to consultation, referral and post-treatment/surgery processes
Monitor and evaluate performance	Monitor performance metrics for every engagement when deployed and continuous evaluation to suit evolving needs and expectations in the healthcare domain.
